# New Houses

**Published:** 1874-01

**Authors:** 


### NEW HOUSES.

THE coincidence of a man's moving into a new house and dying soon after has frequently been a subject of remark, and there is an avoidable cause—the house is moved into before the walls and plaster and the wood are sufficiently dried. Sometimes the cause of death is the poisonous character of the water conveyed through new lead pipes. No water for drinking or cooking purposes should be used in a building supplied with new lead pipes, in whole or in part, for at least one month after the water has been used daily; this gives time for a protecting coating to form on the inner surface of the pipes, when their chemical charge from contact with water generally ceases.

But the damp materials of the house have the most decided effect, especially on persons over fifty years old, or of frail constitutions, whereas if the person were in the full vigor of life and health not even an inconvenience would be experienced.

In building a new house, or on going to live in another locality, where the water supply is not far from the house, it should be ascertained with the utmost certainty that the spring or well is higher than the privies or barn yards

Insidious and fatal forms of decline and typhoid very often result from persons drinking water which is drained from the localities named.

The safest plan, and the only safe place for furnishing dwellings with the most healthful and unobjectionable water, is to have a water tight cistern, and let the water from the roof of the house or barn, or other outhouses be conveyed into it through a box of sand several yards long, this box to rest on a board or cemented bottom and sides, so that no outside water could not get into it.

